# Cytotoxic L-amino-acid oxidases from *Amanita phalloides* and *Clitocybe geotropa* induce caspase-dependent apoptosis

**DOI:** 10.1038/cddiscovery.2016.21

**Published:** 2016-03-21

**Authors:** A Pišlar, J Sabotič, J Šlenc, J Brzin, J Kos

**Affiliations:** 1 Department of Pharmaceutical Biology, Faculty of Pharmacy, University of Ljubljana, Aškerčeva 7, 1000 Ljubljana, Slovenia; 2 Department of Biotechnology, Jožef Stefan Institute, Jamova cesta 39, 1000 Ljubljana, Slovenia

## Abstract

L-amino-acid oxidases (LAO) purified from fungi induce cell death in various mammalian cells including human tumor cell lines. The mechanism, however, remains poorly understood. In this study, we aimed to define a precise mechanism of cell death induced in Jurkat and MCF7 cancer cell lines by ApLAO and CgLAO, LAOs isolated from *Amanita phalloides* and *Clitocybe geotropa*, respectively. Cell death induced by both LAOs is shown to be concentration- and time-dependent, with higher toxic effects in Jurkat cells. LAO activity is required for the cytotoxicity. Detailed study on Jurkat cells further demonstrated that ApLAO and CgLAO both induce the intrinsic mitochondrial pathway of apoptosis, accompanied by a time-dependent depolarization of the mitochondrial membrane through the generation of reactive oxygen species. Treatment with the LAOs resulted in an increased ratio of the expression of proapoptotic Bax to that of antiapoptotic Bcl-2, subsequently leading to the activation of caspase-9 and -3. However, the pancaspase inhibitor, Z-VAD-FMK, did not completely abolish the cell death induced by either ApLAO or CgLAO, suggesting an alternative pathway for LAO-induced apoptosis. Indeed, caspase-8 activity in ApLAO- and CgLAO-treated cells was increased. Further, Fas/FasL (Fas ligand) antagonist caused a slight reduction in toxin-induced cell death, supporting the involvement of ApLAO and CgLAO in death-receptor-mediated apoptosis. These results thus provide new evidence that ApLAO and CgLAO induce apoptosis in Jurkat cells via both the intrinsic and extrinsic pathways, although the significantly higher increase of caspase-9 over caspase-8 activity suggests that it is the intrinsic pathway that is the predominant mode of ApLAO- and CgLAO-induced apoptosis.

Apoptosis is a controlled form of cell death that has an important role in the development and maintenance of higher organisms. Deregulation of apoptosis disrupts the fine balance between cell proliferation and cell death leading to diseases such as cancer.^1^ Apoptosis is defined by several morphological and biochemical hallmarks such as cell shrinkage, membrane blebbing, chromatin condensation and exposure of phosphatidylserine at the plasma membrane.^2^ It is triggered by two main pathways, extrinsic and/or intrinsic. The former, also known as the death receptor pathway, involves the ligation of death receptors such as the tumor necrosis factor receptor and Fas receptor, and results in activation of the initiator caspase-8. The apoptosis signal is propagated by direct cleavage of downstream effector caspases such as caspase-3.^3^ The intrinsic pathway, also known as the mitochondrial pathway, is initiated by the translocation of Bax into the mitochondria. This is followed by dissipation of the mitochondrial membrane potential (Δ*ψ*m) and release of effectors, including cytochrome *c*. The latter triggers the proteolytic activity of caspase-3 and caspase-9 in the cytosol.^4^ Links between the death receptor and mitochondrial apoptotic pathways exist at different levels; however, initiation of each pathway depends on the apoptotic stimuli.^5^

Apoptotic stimuli include the binding of Fas ligand (FasL) with Fas receptor or the binding of other ligands or agonistic antibodies to death receptors,^6–8^ reduced levels of growth factors^9^ and cytotoxic stimuli such as ionizing radiation,^10^ UV irradiation^11^ and anticancer drugs.^1^ In addition, L-amino-acid oxidases, LAOs, have recently been reported to induce apoptosis.^12–16^ LAOs (EC 1.4.3.2) are widely distributed enzymes involved in a variety of basal metabolic pathways. They are also flavoproteins that catalyze oxidative deamination of L-amino acids, with strict stereospecificity, to the corresponding *α*-keto acids via an amino-acid intermediate, producing hydrogen peroxide (H_2_O_2_) and ammonia. Although LAOs have been described from microbial, plant and animal sources, the most extensively studied ones are those from snake venoms. They are a major toxic component of snake venom, comprising up to 10% of total venom protein. LAO activity induces edema and causes hemolytic and hemorrhagic effects; however, it also promotes antimicrobial, antiviral and antiparasitic effects, induces apoptosis of tumor cells and inhibits platelet aggregation. These effects caused by LAO activity are mediated mainly by the production of H_2_O_2_,^17,18^ leading to oxidative stress.^14^ Although the apoptosis-inducing effects of several snake venom LAOs have been studied, with a view to their possible use in cancer therapy, the exact mechanism is still not known. Their lack of cytotoxicity to non-tumorigenic cells and their strong cytotoxicity against various cancer cell lines increases their value for biomedical applications.^17,19–21^

LAOs from sources other than snake venom, mainly microbial, are also increasingly attracting attention.^22^ We have recently shown that fruiting bodies of higher fungi are rich in LAO activities (unpublished results). Among these are two cytotoxic LAOs, toxophallin from *Amanita phalloides* (ApLAO)^15^ and toxovirin from *Amanita virosa*,^23^ both of which possess high toxicity *in vitro* towards the mammalian cells A549, CCL-64, MCF7, T47D, L1210, CEM T4 and Jurkat cells.

In the present study, we aimed to define a precise mechanism for the cell death induced by LAOs toxophallin, from *Amanita phalloides* (ApLAO), and toxogein, from *Clitocybe geotropa* (CgLAO). Similar to ApLAO, the novel purified toxin CgLAO is shown to be cytotoxic to Jurkat and MCF7 cells, and LAO activity is necessary for the toxin-induced cell death and apoptosis. Further, detailed study on Jurkat cells has demonstrated that ApLAO and CgLAO are involved in the multiple signaling pathways triggering apoptosis.

## Results

### Isolation and characterization of ApLAO and CgLAO

The protein fraction exhibiting LAO activity was isolated from fruiting bodies of *A. phalloides* and *C. geotropa* using size-exclusion and ion-exchange chromatographies. SDS-PAGE analysis showed a single band in the *A. phalloides* fraction corresponding to a molecular mass of 72 kDa (Supplementary Figure S1a). The protein toxophallin was isolated by mass spectrometry analysis (ESI-MS) (EMBL-EBI ID: ADA58360; Stasyk *et al*.^15^). Furthermore, LAO activity was confirmed by in-gel detection using L-Leu as the substrate (Supplementary Figure S1b). The *C. geotropa* fraction revealed one prominent band at ~65 kDa on SDS-PAGE (Supplementary Figure S1a) and several bands of LAO activity under non-denaturing conditions (Supplementary Figure S1b), probably as a result of post-translational modifications or of different oligomeric forms. Comparison of the specific activity of the two proteins for oxidation of L-Leu revealed that ApLAO is ~8 times more active than CgLAO.

### ApLAO and CgLAO exert different cytotoxic profiles in Jurkat and MCF7 cells

**Figure 1:**
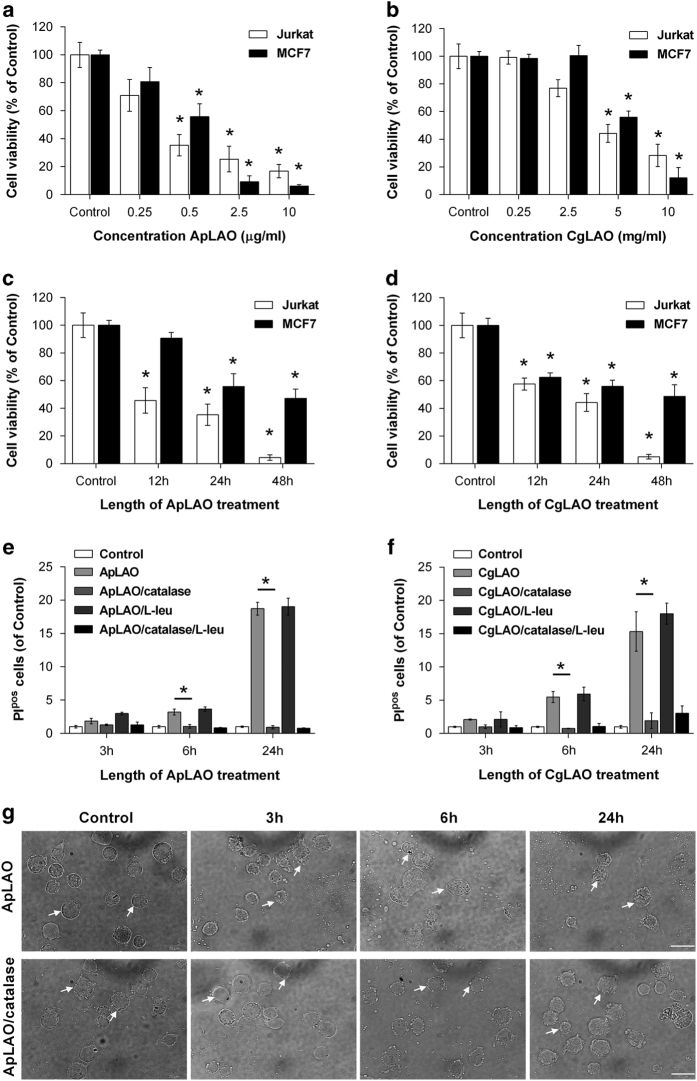
Effects of toxins ApLAO and CgLAO on survival of Jurkat and MCF7 cells. (**a** and **b**) Jurkat cells and MCF7 cells were exposed to the increasing concentrations of ApLAO (0.25–10 *μ*g/ml) and CgLAO (0.25–10 *μ*g/ml). After 24 h of treatment, cell viability was assessed by MTS assay. Results are the means±S.D. of three independent assays. *P<0.05. (**c** and **d**) Jurkat cells and MCF7 cells were exposed to 0.5 *μ*g/ml of ApLAO and 5 *μ*g/ml of CgLAO for the time period indicated, followed by MTS assay. Cells were treated in quadruplicate. Results are the means±S.D. of three independent assays. *P<0.05. (**e** and **f**) Jurkat cells were treated with 0.5 *μ*g/ml ApLAO (left) or 5 *μ*g/ml CgLAO (right) in the absence or presence of catalase (1000 U/ml) and/or L-Leu (790 *μ*g/ml) for the time indicated. They were then harvested and labeled with PI and the percentage of PI^pos^ cells was determined by flow cytometry. Cells were treated in duplicate. Results are the means±S.D. of two independent assays. *P<0.05. (**g**) Representative images of morphological changes of cell membrane integrity (white arrows) following treatment of Jurkat cells with ApLAO (0.5 *μ*g/ml) in the absence or presence of catalase (1000 U/ml) at indicated times. Cells were observed under an inverted phase-contrast microscope. Scale bars=20 *μ*m.

The cytotoxic action of the purified toxins ApLAO and CgLAO was monitored by measuring their effects on T-lymphocyte Jurkat cells and human breast epithelial MCF7 cells. Cells were cultured in complete medium, and then treated with increasing concentrations of ApLAO or CgLAO (0.25–10 *μ*g/ml) at the times indicated. Treatment of the two cell lines with ApLAO for 24 h significantly reduced their viability in a concentration-dependent manner. The 0.5 *μ*g/ml concentration of ApLAO, which reduced the viability of Jurkat cells to 35.4±7.7% and of MCF7 to 55.8±9.2% (Figure 1a), was selected for further study. CgLAO at 5 *μ*g/ml concentration for 24 h reduced the cell viability of Jurkat cells to 44.3±6.4% and of MCF7 cells to 55.9±4.5% of the respective controls (Figure 1b). The reduction of viability of both Jurkat and MCF7 cells was time-dependent; for both oxidases the effect was more pronounced in Jurkat cells (Figures 1c and d).

To establish whether cell death induced by ApLAO and CgLAO is mediated by their oxidase activity, the effect of antioxidant catalase was studied on toxin-treated Jurkat cells. After 3, 6 and 24 h incubation with either ApLAO (0.5 *μ*g/ml) or CgLAO (5 *μ*g/ml), cell membrane integrity was assessed by flow cytometry using propidium iodide (PI). Catalase (1000 U/ml) completely canceled ApLAO- (Figure 1e) and CgLAO- (Figure 1f) induced toxicity. The protective effect of catalase was further demonstrated by morphological changes observed in treated cells (Figure 1g). ApLAO disrupted cell membrane integrity and induced membrane blebbing (white arrows), which was apparent at 3 h and profound at 24 h; however, the presence of catalase maintained the cell membrane integrity. A similar effect on cell membrane integrity was observed for CgLAO (data not shown). To confirm the oxidase activity-mediated cell death, L-Leucine (L-Leu) (790 *μ*g/ml) was used as a substrate for both LAOs (unpublished results). The toxic effect of ApLAO and CgLAO was found to be similar in either the presence or the absence of amino-acid L-Leu (Figures 1e and f).

### ApLAO- and CgLAO-induced apoptosis is mediated by H_2_O_2_

**Figure 2:**
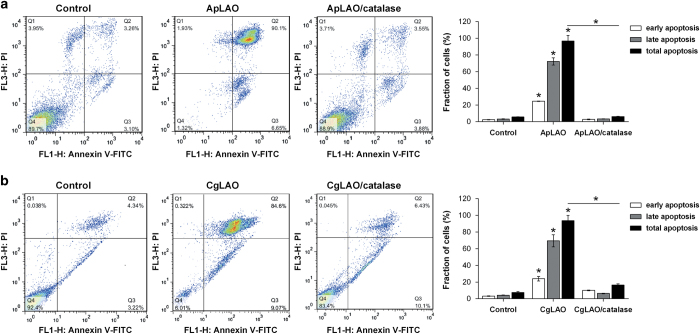
ApLAO- and CgLAO-induced apoptosis in Jurkat cells. (**a** and **b**) Cells were treated with 0.5 *μ*g/ml ApLAO (**a**) and 5 *μ*g/ml CgLAO (**b**) in the absence or presence of catalase (1000 U/ml) for 16 h. Percentages of apoptotic cells were determined by flow cytometry using Annexin V and PI staining. The quadrant threshold was set according to control Jurkat cells and cells treated with toxin, and early apoptotic cells (Annexin V^pos^, PI^neg^) and late apoptotic cells (Annexin V^pos^, PI^pos^) were determined. Graphs (right panels) show quantified analysis and represent the percentage of the fraction of cells showing early apoptosis, late apoptosis and total apoptosis. Results are the means±S.D. of two independent assays. *P<0.05.

The membrane blebbing observed in the presence of both ApLAO and CgLAO is typical of apoptosis.^12^ To examine whether toxin CgLAO is involved in apoptosis – as has been demonstrated for ApLAO^15^ – the mode of cell death was explored by the Anexin V/PI assay using flow cytometry. Apoptosis was determined by detecting the externalized phospholipid phosphatidylserine on the cell surface. Early apoptosis (Annexin V-positive (Annexin V^pos^) and PI-negative (PI^neg^) cells) and late apoptosis (Annexin V^pos^ and PI-positive (PI^pos^) cells) were identified by double staining the cells with Annexin V and PI. Treatment of Jurkat cells with ApLAO (0.5 *μ*g/ml) for 16 h significantly increased both early and late apoptosis, with total apoptotic effects from 5.8 to 96.8% compared with control cells. Nevertheless, the presence of catalase reduced the percentage of Annexin V^pos^ to 6.2% (Figure 2a). The same effect was observed for CgLAO, which increased the total number of apoptotic cells from 7.6 to 93.7% – the presence of catalase decreased the apoptotic effect to 16.5% (Figure 2b).

**Figure 3:**
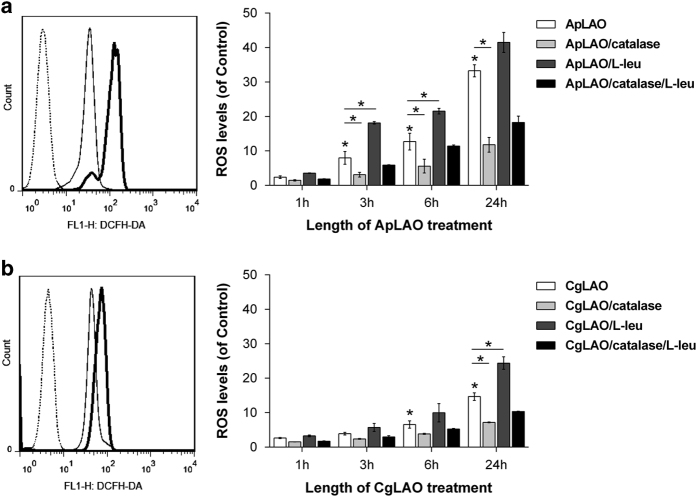
Effects catalase and L-Leu on ApLAO- and CgLAO-induced ROS generation. (**a** and **b**) Jurkat cells were exposed to ApLAO (0.5 *μ*g/ml) (**a**) or CgLAO (5 *μ*g/ml) (**b**) in the absence or presence of catalase (1000 U/ml) and/or L-Leu (790 *μ*g/ml) for the time period indicated. The intracellular ROS levels were measured by flow cytometry using fluorescent DCFH-DA probe. Representative images (left panels) of flow cytometric analysis of ROS generation, where thin dashed line represents control Jurkat cells, thin solid line cells treated with ApLAO (**a**) or CgLAO (**b**) and thick solid line cells treated with toxin in the presence of catalase for 24 h. Graphs (right panels) present flow cytometric analysis of ROS levels as a relative percentage of DCF-positive cells normalized to appropriate control. Results are the means±S.D. of at least two independent assays. *P<0.05.

LAOs are known to generate significant amounts of reactive oxygen species (ROS), creating a state of oxidative stress leading to apoptosis.^14^ Intracellular ROS production was therefore measured using an H_2_DCF-DA probe. Treatment of Jurkat cells with either ApLAO (0.5 *μ*g/ml) or CgLAO (5 *μ*g/ml) significantly increased the generation of ROS in a time-dependent manner, a 33.3-fold increase in a number of DCF-positive cells being observed in cells treated with ApLAO for 24 h (Figure 3a). The effect of CgLAO on ROS generation was less profound; however, on similar treatment for 24 h, CgLAO significantly increased the number of DCF-positive cells to 14.7 times that of control cells (Figure 3b). The presence of catalase (1000 U/ml) in both cases reduced ROS generation in Jurkat cells. Further, the effect of an excess of L-Leu on ROS generation was examined. When L-Leu (790 *μ*g/ml) was added to the culture medium containing ApLAO, ROS generation was enhanced at all the times indicated; however, the presence of both catalase and L-Leu in the culture medium together with ApLAO clearly decreased the number of DCF-positive cells (Figure 3a). A similar effect was also observed in the case of treatment with CgLAO (Figure 3b).

### LAOs trigger caspase-dependent apoptosis in different modes in Jurkat and MCF7 cells

**Figure 4:**
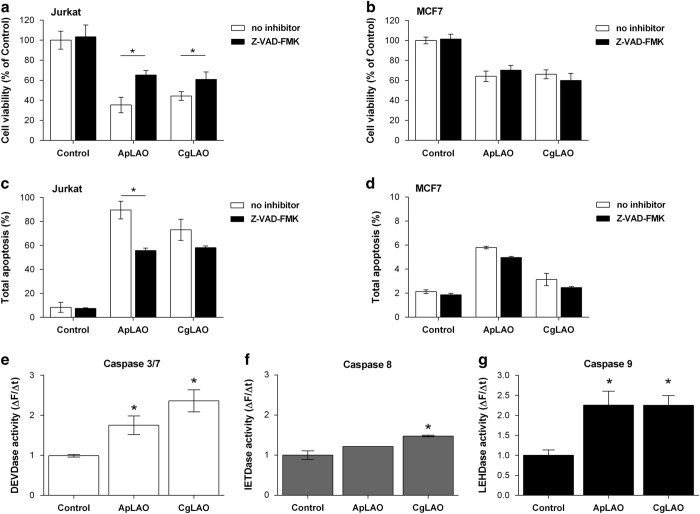
ApLAO- and CgLAO-induced caspase-dependent apoptosis. (**a–d**) Jurkat cells and MCF7 cells were pretreated with pan-specific caspase inhibitor Z-VAD-FMK (10 *μ*M) for 30 min, followed by ApLAO (0.5 *μ*g/ml) and CgLAO (5 *μ*g/ml) treatment. (**a** and **b**) After 24 h, cell viability in Jurkat (**a**) and MCF7 cells (**b**) was assessed by MTS assay. Results are the means±S.D. of three independent assays. *P<0.05. (**c** and **d**) After 16 h of treatment, apoptotic cells were assessed by flow cytometry using Annexin V and PI staining. Graphs show quantified analysis of apoptosis in Jurkat cells (**c**) and MCF7 cells (**d**) and represent the percentage of the fraction of cells showing total apoptosis. Results are the means±S.D. of least two independent assays. *P<0.05. (**e–g**) Jurkat cells were treated with ApLAO (0.5 *μ*g/ml) and CgLAO (5 *μ*g/ml) for 12 h. Caspase activity in cell lysates was determined fluorometrically using the specific substrates for caspase-3/7 (AC-DEVD-AFC) (**e**), caspase-8 (z-IETD-AFC) (**f**) and caspase-9 (Ac-LEHD-AFC) (**g**). The results are presented as changes in fluorescence as a function of time (Δ*F*/Δ*t*). Cells were treated in duplicate. Results are the means±S.D. of at least two independent assays. *P<0.05.

Caspases have a key role in the signaling pathway, by which they are considered as major executers of cell death.^24^ To examine the activation of the caspase cascade, we first pretreated cells with Z-VAD-FMK (10 *μ*M), a broad-spectrum irreversible inhibitor of the caspase family. It partially abolished the cytotoxic effects of ApLAO and of CgLAO in Jurkat cells (Figure 4a), whereas no reduction in cell death was observed in MCF7 cells treated with each of the toxins (Figure 4b). Additionally, pretreatment of Jurkat cells with Z-VAD-FMK reduced the percentage of Annexin V^pos^ cells treated with ApLAO from 96.8±7.3 to 55.8±2.0% and cells treated with CgLAO from 93.7±8.8 to 58.2±1.3% (Figure 4c), indicating that caspases have a critical role in the induction of apoptosis by the studied toxins. However, no significant reduction in apoptosis in the presence of Z-VAD-FMK was observed in MCF7 cells (Figure 4d).

To confirm the suggestion that ApLAO and CgLAO induce caspase-dependent cell death, the enzymatic activity of caspases was measured after cell treatment for the time period indicated. In Jurkat cells treated with ApLAO (0.5 *μ*g/ml), caspase-3/7 activity was increased, peaking at 12 h (Figure 4e). Similarly, treatment of Jurkat cells with CgLAO also led to increased caspase-3/7 activity (Figure 4e). The activity at 12 h was significantly decreased by the presence of catalase (1000 U/ml) for both toxins (data not shown). Activation of caspase-8 and -9 was also examined (Figures 4f and g). Treatment of Jurkat cells with ApLAO and CgLAO for 12 h increased caspase-8 (Figure 4g) as well caspase-9 activity (Figure 4g), the effect being more pronounced in the case of caspase-9. Taken together, these results demonstrate that CgLAO and, likewise, ApLAO induce apoptosis in Jurkat cells in a caspase-dependent manner.

### ApLAO and CgLAO induce mitochondrial abnormalities in Jurkat cells

**Figure 5:**
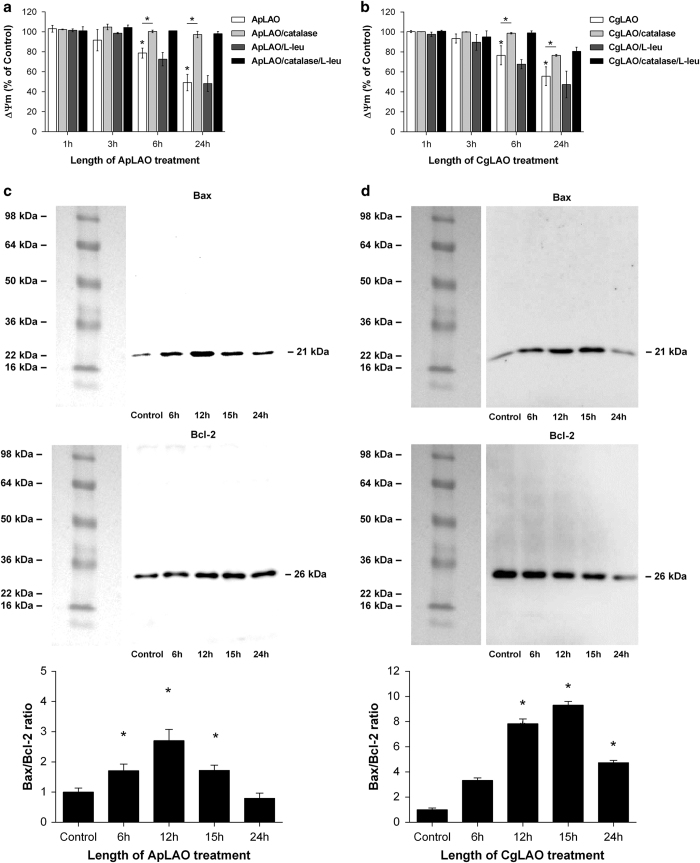
ApLAO- and CgLAO-induced loss of mitochondrial function in Jurkat cells. (**a** and **b**) Cells were exposed to ApLAO (0.5 *μ*g/ml) (**a**) or CgLAO (5 *μ*g/ml) (**b**) in the absence or presence of catalase (1000 U/ml) and/or L-Leu (790 *μ*g/ml) for the time period indicated. Mitochondrial transmembrane potential (Δ*ψ*m) was then measured by flow cytometry using mitochondria-sensitive CMXRos dye. Cells were treated in duplicate. Results are the means±S.D. of two independent assays. *P<0.05. (**c** and **d**) Western blots showing the effect of ApLAO (0.5 *μ*g/ml) (**c**) and CgLAO (5 *μ*g/ml) (**d**) treatment at the time period indicated on the protein levels of Bax (upper panel) and Bcl-2 (lower panel) in Jurkat cells. The bar plot shows densitometric analysis of protein expression and represents the ratio of Bax to Bcl-2 expression (Bax/Bcl-2) relative to that in control cells. Results are the means±S.D. of two independent assays. *P<0.05.

It is well known that damage of mitochondria has a very important role in the intrinsic pathway of apoptosis.^25^ Alteration in the Δ*ψ*m was therefore evaluated by flow cytometric analysis, using the mitochondria-sensitive MitoTracker Red CMXROS dye, in cells treated with ApLAO (0.5 *μ*g/ml) or CgLAO (5 *μ*g/ml) in the absence or presence of catalase (1000 U/ml). Significant dissipation Δ*ψ*m appeared after 6 h treatment with ApLAO (Figure 5a) and with CgLAO (Figure 5b), increasing further with time of incubation. After 24 h, in the case of ApLAO treatment, Δ*ψ*m was reduced to 49.2±6.2% of the value for control cells. Following CgLAO treatment, Δ*ψ*m was reduced to 55.7±8.3% that of control cells. This reduction, however, was reversed in the presence of catalase, when mitochondrial function after 24 h of incubation with ApLAO recovered to 71.4±5.1% of the control value and, with CgLAO, to 76.7±0.9% (Figures 5a and b). When an amino-acid, L-Leu, was used, no significant changes in the ApLAO- or CgLAO-increased loss of vΔ*ψ*m were observed (Figures 5a and b).

Given the ability of both toxins to induce mitochondrial damage through dissipation of Δ*ψ*m, we also assessed their effects on the expression of proapoptotic Bax and antiapoptotic Bcl-2 in Jurkat cells. Western blot analysis showed significant increases in the level of Bax protein in cells treated with ApLAO (0.5 *μ*g/ml) (Figure 5c) and with CgLAO (5 *μ*g/ml) (Figure 5d). Similarly, ApLAO induced a slight increase in the level of Bcl-2 protein (Figure 5c), whereas CgLAO decreased the expression of Bcl-2 protein in a time-dependent manner (Figure 5d). Nevertheless, treatment of cells with each of the LAOs resulted in increased Bax/Bcl-2 ratios, indicating mitochondria-dependent induced apoptosis.

### LAOs interfere in death receptor-mediated apoptosis

**Figure 6:**
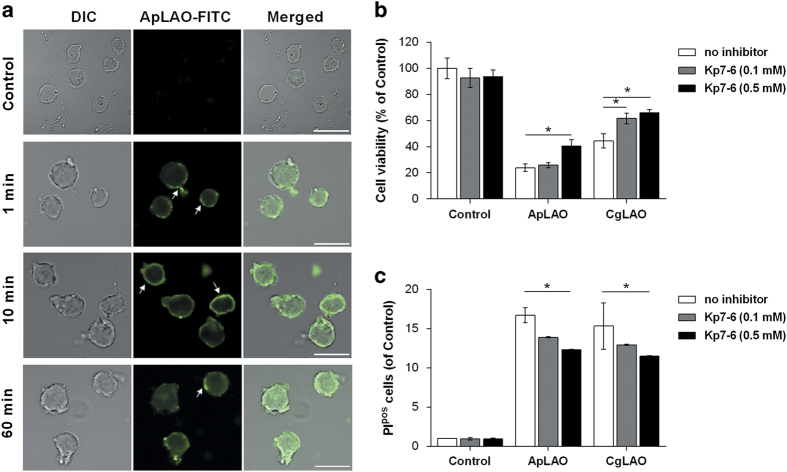
Effects of ApLAO and CgLAO in death receptor-mediated apoptosis of Jurkat cells. (**a**) Representative images of the localization of ApLAO conjugated with FITC (ApLAO-FITC; green fluorescence) in control Jurkat cells and in cells treated with ApLAO-FITC for 1, 10 and 60 min (white arrows), as analyzed by fluorescence microscopy. Scale bars=20 *μ*m (**b** and **c**) Jurkat cells were pretreated with Fas/FasL inhibitor Kp7-6 (0.1–0.5 mM) for 1 h, followed by ApLAO (0.5 *μ*g/ml) or CgLAO (5 *μ*g/ml) treatment. After 24 h, cell viability was assessed by MTS assay (**b**). Cells were treated in quadruplicate. Results are the means±S.D. of three independent assays. *P<0.05. Cytotoxicity was assessed by staining cells with PI and the percentage of PI^pos^ cells was determined by flow cytometry (**c**). Cells were treated in duplicate. Results are the means±S.D. of two independent assays. *P<0.05.

The Fas receptor is a death receptor on the surface of cells that leads to the extrinsic apoptotic pathway, through caspase-8 activation.^26^ As an increase in caspase-8 activation was observed in cells treated with LAOs, we further investigated whether the extrinsic pathway of apoptosis is triggered. To address this question, ApLAO protein was conjugated with a fluorescent molecule FITC (ApLAO-FITC). Exposure of Jurkat cells to ApLAO-FITC showed the periplasmic membrane localization of ApLAO after 1 and 10 min of treatment, whereas 1 h treatment resulted in morphological changes and reduced fluorescence of the conjugated protein (Figure 6a). Further, the ability of LAO to induce cell death by association with death receptor was investigated. Jurkat cells were pretreated with Fas/FasL antagonist Kp7-6 (0.5 mM) for 1 h and then treated with ApLAO (0.5 *μ*g/ml) or CgLAO (5 *μ*/ml) for an additional 24 h. Fas/FasL antagonist had a small impact on the viability of ApLAO- and CgLAO-reduced cells (Figure 6b). Furthermore, ApLAO- and CgLAO-induced damage of cell membrane integrity, as assessed by flow cytometry, was reduced in the presence of 0.5 mM Kp7-6 (Figure 6c), suggesting that Fas receptor has some role in LAO-induced cell death.

## Discussion

The toxic effects of a toxogein isolated from the fruiting bodies of the edible trooping funnel *C. geotropa* (CgLAO) have been studied in comparison with those of toxophallin isolated from the death cap *A. phalloides* (ApLAO), using Jurkat and MCF7 cancer cell lines. CgLAO has been shown to exert a cytotoxic effect similar to that caused by ApLAO, and that LAO activity is required for toxin-induced cell death and apoptosis. Study in more detail of the mechanism of LAO-induced toxicity in Jurkat cells revealed that both purified toxins ApLAO and CgLAO trigger caspase-dependent apoptosis.

Several studies showed that LAOs exhibits cell-type-specific toxicity in a concentration-dependent manner, causing either apoptosis or necrosis.^12,23,27,28^ In our study, treatment of Jurkat and MCF7 cells with ApLAO and CgLAO triggered cell death in a time-dependent as well as concentration-dependent manner. Results showed that, in general, ApLAO and CgLAO act similarly towards used types of cancer cells: in both cases human Jurkat T cells were more sensitive and breast cancer MCF7 cells were more resistant to the action of these toxic proteins. The latter coincide with previous studies where Jurkat cells were also shown to be more sensitive to toxic effects of the fungal LAOs towards other cell lines similar to MCF7.^15,23^

LAO-mediated cell death is a consequence of protracted exposure to high levels of H_2_O_2_, an ROS. It is well established that, under conditions of severe oxidative stress, cell membrane integrity is compromised, leading to membrane blebbing, cell shrinkage and opening of the permeability transition pores resulting in cell lysis.^2^ Murakawa *et al.*^12^ showed that apoptosis-inducing protein (AIP), which also possess LAO activity, induce H_2_O_2_-mediated and rapid apoptosis in HL-60 cells. The latter effect was clearly inhibited by an antioxidant catalase, most probably as a result of scavenging the oxidative catalysis product H_2_O_2_. Indeed, in our study the removal of H_2_O_2_, by the addition of catalase to the medium, inhibited morphological changes and cell death in Jurkat cells, mediated by each of the toxins. In addition, both early and late apoptosis were completely diminished in the presence of catalase. Nevertheless, it has also been reported that LAO could induce H_2_O_2_-independent apoptosis even in the presence of catalase, although this delayed apoptosis was completely abolished by the addition of the specific amino acid to the medium.^12^ Interestingly, we observed that addition of L-Leu, which is the best substrate of ApLAO and CgLAO, did not inhibit cell death but resulted in slight increase of LAO-mediated toxicity, probably due to pronounced oxidative action.

The cytotoxic activity of snake venom LAOs has been linked to the production of H_2_O_2_, which accumulates on the cell surface and triggers oxidative stress in cancer cells leading to apoptosis.^13,29^ An increasing accumulation of intracellular H_2_O_2_ was found in ApLAO- or CgLAO-treated Jurkat cells, which was probably related to the LAO activity. Moreover, catalase, the ROS scavenger, significantly reduced intracellular ROS as well as preventing apoptosis, whereas, on the contrary, an excess of L-Leu additionally increased intracellular ROS levels that were also reduced in the presence of catalase. Similarly, the addition of catalase, which neutralizes the oxidative catalysis product H_2_O_2_, inhibited apoptosis of several cell lines induced by LAO Apoxin-I from rattlesnake venom.^29^ Apoptosis induced by AIP in HL-60 cells was also shown to be inhibited by catalase. However, in this case only the rapid H_2_O_2_-dependent induction was inhibited and delayed apoptosis of HL-60 cells was observed in the presence of catalase. The protection afforded by the antioxidant against the induction of cell death by ApLAO or CgLAO suggests that ROS may be involved in the LAO-mediated apoptotic process. Nevertheless, recent studies show that, apart from the production of H_2_O_2_-stimulated cell apoptosis, LAOs may induce caspase-dependent apoptosis.^16,21,30^ This has been demonstrated for snake venom LAOs, whereas the apoptosis triggered in mammalian cells with mushroom LAOs such as toxophallin did not depend on the activation of the caspase cascade.^15^ Here, we have shown, however, that an inhibitor of the caspase family Z-VAD-FMK partially protected cells from a cytotoxic and apoptotic insult, indicating that, under these conditions, caspases are possible death effectors. This effect was evident only in Jurkat cells, whereas no significant changes in the presence of Z-VAD-FMK were observed in LAO-induced apoptosis and cell death in MCF7 cells, which is likely due to more increased necrosis than apoptosis in MCF7 cells in response to toxic effect of LAOs, indicating a cell-type-dependent mode of action of LAOs. Indeed, it has been shown that tumor cells with different origins have different threshold to apoptosis. Hematopoietic cells such as Jurkat cells have a lower threshold to oxidative stress and induced apoptosis than non-hematopoietic cells such as MCF7 as a result of increased accumulation of H_2_O_2_ in hematopoietic cells.^31^ However, the presence of LAO-specific receptors or targets in different cell lines that are involved in the induction of apoptosis or cell death remain unknown and need to be revealed.

Snake venom LAOs induce caspase-dependent apoptosis via intrinsic (mitochondrial) or extrinsic (death-receptor) pathway.^16,21,30^ The intrinsic pathway of apoptosis is associated with depolarization of the mitochondrial membrane through generation of ROS, consequently leading to the release of cytochrome *c* from the mitochondria to the cytosol, with the activation of caspase-9 and -3.^32,33^ We demonstrated that caspase-3 and -9 are activated by either ApLAO or CgLAO in a time-dependent manner in Jurkat cells. Furthermore, LAO-induced cell death was accompanied by dissipation of Δ*ψ*m and was reversed in the presence of catalase, whereas the addition of L-Leu to the medium had no significant effect on Δ*ψ*m. However, so far results indicate that toxin-induced apoptosis in Jurkat cells is mediated through the intrinsic pathway. This pathway is regulated by the Bcl-2 protein family, mainly by the antiapoptotic protein Bcl-2 and proapoptotic protein Bax.^34^ The ratio of Bax to Bcl-2 may be a better marker of apoptosis than just their levels, as it represents the balance between the pro- and antiapoptotic proteins levels. We showed that the Bax/Bcl-2 ratio increased in both ApLAO and CgLAO treatment in a time-dependent manner, which additionally confirms the involvement of LAOs in the intrinsic pathway of apoptosis.

On the other hand, the extrinsic pathway of apoptosis is triggered by the binding of ligands with their cognate death receptors, such as Fas/FasL.^35^ Consequently, through a series of events, procaspase-8 is activated to caspase-8, leading to caspase-3 activation and apoptosis.^36^ Involvement of the death receptor-mediated pathway of apoptosis of Jurkat cells by ApLAo and CgLAO was indicated by increased caspase-8 activity. Moreover, immunofluorescence of FITC-conjugated ApLAO revealed periplasma localization of the protein, and, further, using a Fas/FasL antagonist showed the involvement of ApLAO, as well as CgLAO, in death-receptor-mediated apoptosis. Localization to the plasma membrane has been shown to be important for the antibacterial activity of several LAOs, and binding of plasma membrane proteins probably contributes to the specificity of LAOs for distinct bacteria.^37–39^ The antimicrobial activity of LAO is likely associated with H_2_O_2_ formation as it is significantly decreased in the presence of H_2_O_2_ scavengers such as catalase.^22^ Similar mechanism has been observed in our study, where apoptosis increased by both LAOs diminished in the presence of catalase. Nevertheless, so far, caspase-independent apoptosis activation has been observed for fungal LAOs in different cell lines; however, here we showed for the first time that the activation of both intrinsic and extrinsic pathways is triggered.

**Figure 7:**
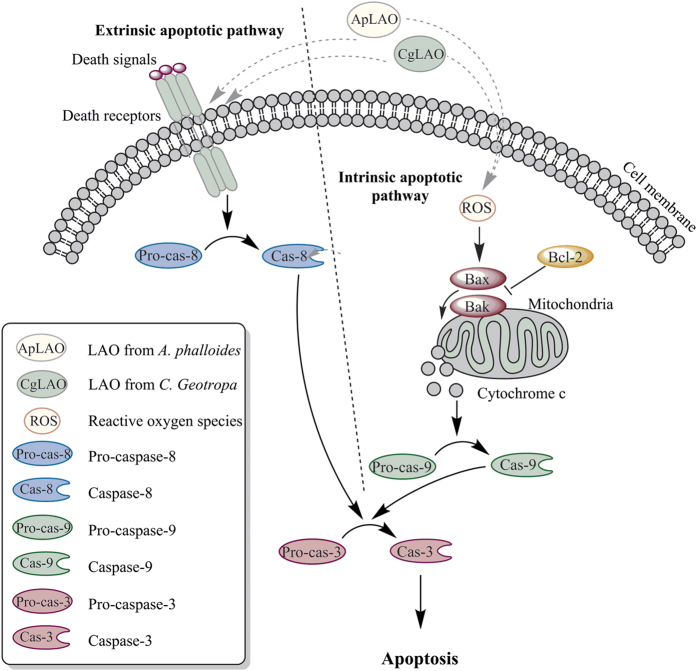
Proposed molecular mechanism of the apoptotic action of LAOs in Jurkat cells. ApLAO and CgLAO can induce apoptosis by both the intrinsic and extrinsic pathways. The intrinsic mitochondrial pathway is triggered by mitochondrial dysfunctions, including generation of intracellular ROS, decreased Δ*ψ*m, increased Bax/Bcl-2 ratio and caspase-9 activation, leading to increased caspase-3 and consequently to apoptosis. Extrinsic, death receptor-mediated apoptosis is triggered by caspase-8 activation.

In conclusion, as summarized in Figure 7, our results provide a new mechanism of cell death induced by the fungal ApLAO and CgLAO. Our study suggests that these LAOs induce apoptosis in Jurkat cells via both intrinsic and extrinsic pathways, although the mitochondrial intrinsic pathway predominates. Emerging evidence demonstrates that snake venom LAOs can disturb the cell cycle status of cancer cells, induce apoptosis and effectively inhibit tumor growth, thereby having the potential to be developed into an antitumor drug.^40,41^ However, most snake venom LAOs are thermolabile proteins, and not appropriate for drug formulations; therefore, new sources of stable LAOs, such as fungal LAOs, may significantly strengthen their applicability as anticancer agents.

## Materials and Methods

### Isolation and purification of cytotoxic proteins from *A. phalloides* and *C. geotropa*

Fruiting bodies of death cap *A. phalloides* and trooping funnel *C. geotropa* were collected from their natural habitat in Slovenia and frozen at −20 °C. Crude mushroom extracts were prepared by homogenizing the thawed fruiting bodies and centrifuging at 16 000×*g* for 5 min to remove insoluble material. The extract was concentrated by ultrafiltration (3 kDa cutoff) and applied to size-exclusion chromatography on Sephacryl S-200 equilibrated with 0.02 M Tris-HCl, pH 7.5, and 0.3 M NaCl. Fractions exhibiting LAO activity were pooled, concentrated by ultrafiltration and dialyzed against 50 mM phosphate buffer, pH 6.8, containing 0.85 M ammonium sulfate, and then applied to phenyl-Sepharose hydrophobic interaction chromatography. Bound proteins were eluted with a linear gradient of ammonium sulfate from 0.85 to 0 M in 50 mM phosphate buffer, pH 6.8, followed by a gradient of 0–20% ethanol in the same buffer. Fractions showing LAO activity were pooled and concentrated by ultrafiltration. Protein fractions were analyzed by SDS-PAGE and silver staining and by in-gel detection of LAO activity. The identity of ApLAO (toxophallin) was confirmed by peptide mass fingerprinting (ESI-MS/MS) of the trypsin-digested band excised from SDS-PAGE.

### LAO activity assays

LAO activity in fractions was assayed as described.^42^ The activity was assayed in microplates at 37 °C. Ten microliters of sample was mixed with 90 *μ*l of the substrate reaction mixture in 0.1 M Bis-Tris, pH 5.5, together with 5 mM L-Leu, 2 mM *o*-phenylenediamine and 0.81 U/ml horseradish peroxidase. After termination of the reaction by adding 50 *μ*l of 2M H_2_SO_4_, absorbance was measured at 492 nm using 630 nm as a reference wavelength. Alternatively, LAO activity was analyzed by in-gel detection as described (unpublished results). Samples prepared in the sample buffer without boiling were subjected to SDS-PAGE and bands with LAO activity developed after incubation, at room temperature in the dark, for 1 h in a reaction mixture containing 5 mM L-Leu, 1 mM *o*-phenylenediamine and 0.5 U/ml horseradish peroxidase in 0.1 M Bis-Tris, pH 5.5. The reaction was stopped by adding 2 M H_2_SO_4_ and the results documented with an image scanner.

### Cell cultures

Jurkat T lymphocytes were purchased from American Type Culture Collection (TIB-152; ATCC, Manassas, VA, USA). They were grown in RPMI-1640 medium (Sigma, St Louis, MO, USA) supplemented with 5% (v/v) fetal bovine serum (HyClone, Logan, UT, USA), 2 mM L-glutamine, 50 U/ml penicillin and 50 *μ*g/ml streptomycin (Sigma). The cells were maintained at 37 °C in a humidified atmosphere containing 95% air and 5% CO_2_. MCF7 human breast epithelial cell line was purchased from ATCC (HTB-22). MCF7 cells were grown in DMEM/F12 (1 : 1) medium supplemented with 5% (v/v) fetal bovine serum (HyClone), 1 *μ*g/ml insulin, 0.5 *μ*g/ml hydrocortisone, 50 ng/ml epidermal growth factor, 2 mM L-glutamine, 50 U/ml penicillin and 50 *μ*g/ml streptomycin (Sigma). The cells were maintained at 37 °C in a humidified atmosphere containing 95% air and 5% CO_2_, and grown to 80% confluence.^43^ For the experiments, purified ApLAO and CgLAO were diluted in the culture medium. Cells treated with the culture medium without ApLAO or CgLAO were used as a negative control.

### Cell viability assay

Cell viability was evaluated by MTS assay as described.^43^ Jurkat cells (5×10^4^/well) were seeded into a 96-well plate in quadruplicate in both the absence and presence of the inhibitors Z-VAD-fmk (10 *μ*M) (Bachem, Bubendorf, Switzerland) or Kp7-6 (0.1–0.5 mM) (Calbiochem, Billerica, MA, USA) in the culture medium. After an appropriate time of incubation, cells were treated with either ApLAO or CgLAO at different concentrations (0.25–10 *μ*g/ml) for 12–48 h). When required, catalase (1000 U/ml) (Sigma) and/or L-Leu (790 *μ*g/ml) (Sigma) were added to the culture medium containing ApLAO or CgLAO. MCF7 cells (2×10^4^/well) were seeded into a 96-well plate in quadruplicate. The next day, cells were treated as described above for Jurkat cells. Cell viability was assessed using the CellTiter 96 Aqueous One Solution Cell Proliferation Assay (Promega, Madison, WI, USA), in accordance with the manufacturer’s instructions. Absorbance was measured with an automatic microplate reader (Tecan Safire2; Tecan Group Ltd, Männendorf, Switzerland) at a wavelength of 492 nm. Results are presented as percentages of the corresponding vehicle treatment (control cells).

### Assessment of cytotoxicity

The cytotoxicity of the purified toxins was confirmed by flow cytometry analysis using PI (Molecular Probes, Eugene, OR, USA). PI does not cross the cell membrane but stains DNA in cells when the cell membrane is disintegrated. Jurkat cells were seeded in duplicate into a 24-well culture plate (2×10^5^/well) and treated with ApLAO (0.5 *μ*g/ml) or CgLAO (5 *μ*g/ml) in the absence or presence of catalase (1000 U/ml), with or without L-Leu (790 *μ*g/ml). When required, Kp7-6 antagonist (0.1–0.5 mM) was added to the culture medium 1 h before toxin treatment. After 24 h of incubation, cells were washed with prewarmed phosphate-buffered saline (PBS) and further stained with PI solution (30 *μ*M) for 15 min at 37 °C. Cells were then analyzed for cytotoxicity by flow cytometry on FACS Calibur (BD Bioscience, San Jose, CA, USA). The percentage of PI^pos^ cells was evaluated using the FlowJo software (FlowJO, Ashland, OR, USA). The results are presented as relative fold increase of PI^pos^ over the corresponding vehicle treatment (control cells).

### Analysis of morphological changes

Morphological changes, including membrane blebbing, were observed using an inverted microscope. Jurkat cells were seeded in duplicate into a 24-well culture plate (2×10^5^/well) and treated with ApLAO (0.5 *μ*g/ml) in the absence or presence of catalase (1000 U/ml). After 3, 6 and 24 h of incubation, cells were cytospinned on the slides for 6 min at 1300 r.p.m., and then fixed in 10% (w/v) formalin in PBS (pH 7.4) for 15 min at room temperature. After washing with PBS, the Prolong Antifade Kit (Molecular Probes) was mounted on dry slides and allowed to dry overnight at 4 °C. Images were recorded using an Olympus IX 81 motorized inverted microscope (Olympus, Center Valley, PA, USA) with the CellR software (Olympus).

### Detection of apoptosis

Apoptosis was detected and quantified using an Annexin-FITC Apoptosis Detection Kit (Sigma), in accordance with the manufacturer’s instructions. Jurkat and MCF7 cells were cultured in a 24-well plate (2×10^5^/well) and treated with ApLAO (0.5 *μ*g/ml) or CgLAO (5 *μ*g/ml) in the absence or presence of catalase (1000 U/ml) for 16 h. When required, Z-VAD-FMK inhibitor (10 *μ*M) was added to the culture medium 30 min before toxin treatment. After treatment, cells were washed with cold PBS and resuspended in 500 *μ*l of binding buffer. FITC-labeled Annexin V (5 *μ*l) and PI (10 *μ*l) were added to the cells and incubated for 15 min in the dark. Cell apoptosis was analyzed using a FACS Calibur flow cytometer (BD Bioscience). The percentage of apoptotic cells (Annexin V^pos^) was evaluated using the FlowJo software and the results were presented as the percentage of Annexin V^pos^ corresponding to vehicle treatment (control cells).

### Determination of the generation of ROS

Intracellular ROS generation was determined by staining cells with the dichlorofluorescein diacetate (DCFH-DA) probe (Sigma), using flow cytometry. Jurkat cells were washed with PBS and incubated with 10 *μ*M of DCFH-DA in prewarmed PBS for 20 min at 37 °C. The cells were then washed again with PBS, resuspended in the culture medium and seeded into a 24-well culture plate in duplicate (2×10^5^/well). After 15 min at 37 °C, cells were treated with ApLAO (0.5 *μ*g/ml) or CgLAO (5 *μ*g/ml) in the absence or presence of catalase (1000 U/ml) and/or L-Leu (790 *μ*g/ml) for the time period indicated. The cells were then analyzed by a FACS Calibur flow cytometer (BD Bioscience). The intensity of DCFH-DA fluorescence of the cells served as a measure of ROS generation. Data were analyzed using the FlowJo software and results are presented as fold increase of ROS generation relative to that of the corresponding vehicle treatment (control cells).

### Caspase activity assay

The activities of caspase-3/7, -8 and -9 were measured in total cell lysates of Jurkat cells (1×10^6^/ml) treated with ApLAO (0.5 *μ*g/ml) or CgLAO (5 *μ*g/ml), in the absence or presence of catalase (1000 U/ml) as required and for the time period indicated, using an appropriate fluorescent substrate.^44^ Ac-DEVD-AFC (Bachem) was used as a substrate for caspase-3/7, Ac-IETD-AFC (Bachem) for caspase-8 and Ac-LEHD-AFC (Bachem) for caspase-9. Fluorescence was monitored continuously for 30 min using a fluorescence microplate reader (Tecan Safire2) at an excitation wavelength of 405 nm and emission wavelength of 535 nm. Results are presented as change in fluorescence as a function of time.

### Determination of the reduction of Δ*ψ*m

Reduction of Δ*ψ*m was determined by staining cells with MitoTracker dye (MitoTracker Red CMXRos; Molecular Probes, Eugene, OR, USA) using flow cytometry. Jurkat cells were seeded into a 24-well culture plate (2×10^5^/well) and treated with ApLAO (0.5 *μ*g/ml) or CgLAO (5 *μ*g/ml) in the absence or presence of catalase (1000 U/ml) and/or L-Leu (790 *μ*g/ml) for the time period indicated. Cells were then washed with PBS and resuspended in prewarmed culture medium containing 300 nM of MitoTracker dye. After a 30-min incubation at 37 °C, the cells were washed again with PBS, and then analyzed by a FACS Calibur flow cytometer (BD Bioscience). The intensity of red fluorescence of the cells serves as a measure of Δ*ψ*m. Data were analyzed by the FlowJo software and results are presented as a percentage of Δ*ψ*m relative to control set to 100% Δ*ψ*m.

### Western blot analysis of proteins

For protein determination in cell lysates, Jurkat cells were seeded into a 6-well culture plate (5×10^6^/well) and treated with ApLAO (0.5 *μ*g/ml) or CgLAO (5 *μ*g/ml) for the time period indicated. Cells were then harvested in cell lysis buffer (50 mM HEPES, pH 6.5, 150 mM NaCl, 1 mM EDTA, 1% Triton X-100) supplemented with a cocktail of protease and phosphatase inhibitors (Molecular Probes), and then incubated for 30 min on ice. Protein determination and western blotting were performed as described.^45^ In western blotting, the following primary antibodies were applied: anti-Bax (1 : 1000; Cell Signaling, Denvers, MA, USA) and anti-Bcl-2 (1 : 1000; Cell Signaling). Signals from anti-rabbit HRP-conjugated secondary antibodies (1 : 5000; Merck Millipore, Billerica, MA, USA) were visualized by an Enhanced Chemiluminescence Detection Kit (Thermo Scientific, Waltham, MA, USA). Band intensities were quantified using the Gene Tools software (Sygene, Cambridge, UK) and expressed as values relative to those of control.

### Immunofluorescence staining

The subcellular localization of ApLAO was determined by the fluorescent molecule FITC. FITC (3 mg) was added to the purified ApLAO in 0.1 M Na carbonate buffer, pH 11, and incubated 3 h at room temperature with constant stirring. Unbound FITC was removed by dialysis against PBS supplemented with 5 g/l activated carbon, followed by dialysis against PBS in the dark. FITC-labeled ApLAO was concentrated and filter-sterilized. Jurkat cells were seeded into a 24-well culture plate in duplicate (2×10^5^/well) and treated with the ApLAO-FITC (5 *μ*g/ml) for 1, 10 and 60 min. The cells were then cytospun on the slides for 6 min at 1300 r.p.m. and fixed in 10% (w/v) formalin in PBS (pH 7.4) for 15 min at room temperature. After washing with PBS, Prolong Antifade Kit (Molecular Probes) was mounted on dried slides and allowed to dry overnight at 4 °C. A Carl Zeiss LSM 710 confocal microscope (Carl Zeiss, Oberkochen, Germany) with ZEN 2011 image software (Carl Zeiss) was used for fluorescence microscopy.

### Statistical analysis

Results shown are representative of at least two independent experiments, each performed in, at least, duplicate, and are presented as means±S.D. Student’s *t*-test was used for statistical evaluation when two sets of values were compared; *P*<0.05 was considered to be statistically significant.

## Abbreviations

Δ*ψ*m: mitochondrial membrane potential
AIP: apoptosis-inducing protein
ApLAO: LAO from *Amanita phalloides*
CgLAO: LAO from *Clitocybe geotropa*
FasL: Fas ligand
L-Leu: L-leucine
LAO: L-amino-acid oxidase
ROS: reactive oxygen species.

## References

[bib1] Fesik SW. Promoting apoptosis as a strategy for cancer drug discovery. Nat Rev Cancer 2005; 5: 876–885. 16239906 10.1038/nrc1736

[bib2] Elmore S. Apoptosis: a review of programmed cell death. Toxicol Pathol 2007; 35: 495–516. 17562483 10.1080/01926230701320337 PMC2117903

[bib3] Walczak H, Krammer PH. The CD95 (APO-1/Fas) and the TRAIL (APO-2L) apoptosis systems. Exp Cell Res 2000; 256: 58–66. 10739652 10.1006/excr.2000.4840

[bib4] Eldering E, Mackus WJ, Derks IA, Evers LM, Beuling E, Teeling P et al. Apoptosis via the B cell antigen receptor requires Bax translocation and involves mitochondrial depolarization, cytochrome *C* release, and caspase-9 activation. Eur J Immunol 2004; 34: 1950–1960. 15214043 10.1002/eji.200324817

[bib5] Fulda S, Debatin KM. Extrinsic versus intrinsic apoptosis pathways in anticancer chemotherapy. Oncogene 2006; 25: 4798–4811. 16892092 10.1038/sj.onc.1209608

[bib6] Trauth BC, Klas C, Peters AM, Matzku S, Moller P, Falk W et al. Monoclonal antibody-mediated tumor regression by induction of apoptosis. Science 1989; 245: 301–305. 2787530 10.1126/science.2787530

[bib7] Yonehara S, Ishii A, Yonehara M. A cell-killing monoclonal antibody (anti-Fas) to a cell surface antigen co-downregulated with the receptor of tumor necrosis factor. J Exp Med 1989; 169: 1747–1756. 2469768 10.1084/jem.169.5.1747 PMC2189304

[bib8] Itoh N, Yonehara S, Ishii A, Yonehara M, Mizushima S, Sameshima M et al. The polypeptide encoded by the cDNA for human cell surface antigen Fas can mediate apoptosis. Cell 1991; 66: 233–243. 1713127 10.1016/0092-8674(91)90614-5

[bib9] Batistatou A, Greene LA. Aurintricarboxylic acid rescues PC12 cells and sympathetic neurons from cell death caused by nerve growth factor deprivation: correlation with suppression of endonuclease activity. J Cell Biol 1991; 115: 461–471. 1655805 10.1083/jcb.115.2.461 PMC2289153

[bib10] Marini P, Belka C. Death receptor ligands: new strategies for combined treatment with ionizing radiation. Curr Med Chem Anticancer Agents 2003; 3: 334–342. 12871079 10.2174/1568011033482297

[bib11] Pustisek N, Situm M. UV-radiation, apoptosis and skin. Coll Antropol 2011; 35: 339–341. 22220467

[bib12] Murakawa M, Jung SK, Iijima K, Yonehara S. Apoptosis-inducing protein, AIP, from parasite-infected fish induces apoptosis in mammalian cells by two different molecular mechanisms. Cell Death Differ 2001; 8: 298–307. 11319613 10.1038/sj.cdd.4400811

[bib13] Ande SR, Kommoju PR, Draxl S, Murkovic M, Macheroux P, Ghisla S et al. Mechanisms of cell death induction by L-amino acid oxidase, a major component of ophidian venom. Apoptosis 2006; 11: 1439–1451. 16770529 10.1007/s10495-006-7959-9

[bib14] Zhang L, Wei LJ. ACTX-8, a cytotoxic L-amino acid oxidase isolated from *Agkistrodon acutus* snake venom, induces apoptosis in Hela cervical cancer cells. Life Sci 2007; 80: 1189–1197. 17275856 10.1016/j.lfs.2006.12.024

[bib15] Stasyk T, Lutsik-Kordovsky M, Wernstedt C, Antonyuk V, Klyuchivska O, Souchelnytskyi S et al. A new highly toxic protein isolated from the death cap Amanita phalloides is an L-amino acid oxidase. FEBS J 2010; 277: 1260–1269. 20121947 10.1111/j.1742-4658.2010.07557.x

[bib16] Mukherjee AK, Saviola AJ, Burns PD, Mackessy SP. Apoptosis induction in human breast cancer (MCF-7) cells by a novel venom L-amino acid oxidase (Rusvinoxidase) is independent of its enzymatic activity and is accompanied by caspase-7 activation and reactive oxygen species production. Apoptosis 2015; 20: 1358–1372. 26319994 10.1007/s10495-015-1157-6

[bib17] Guo C, Liu S, Yao Y, Zhang Q, Sun MZ. Past decade study of snake venom L-amino acid oxidase. Toxicon 2012; 60: 302–311. 22579637 10.1016/j.toxicon.2012.05.001

[bib18] Lukasheva EV, Efremova AA, Treshchalina EM, Arinbasarova A, Medentsev AG, Berezov TT. L-Amino acid oxidases: properties and molecular mechanisms of action. Biomed Khim 2012; 58: 372–384. 23413682 10.18097/pbmc20125804372

[bib19] Lee ML, Fung SY, Chung I, Pailoor J, Cheah SH, Tan NH. King cobra (*Ophiophagus hannah*) venom L-amino acid oxidase induces apoptosis in PC-3 cells and suppresses PC-3 solid tumor growth in a tumor xenograft mouse model. Int J Med Sci 2014; 11: 593–601. 24782648 10.7150/ijms.8096 PMC4003544

[bib20] Izidoro LF, Sobrinho JC, Mendes MM, Costa TR, Grabner AN, Rodrigues VM et al. Snake venom L-amino acid oxidases: trends in pharmacology and biochemistry. Biomed Res Int 2014; 2014: 196754. 24738050 10.1155/2014/196754 PMC3971498

[bib21] Burin SM, Ayres LR, Neves RP, Ambrosio L, de Morais FR, Dias-Baruffi M et al. L-Amino acid oxidase isolated from *Bothrops pirajai* induces apoptosis in BCR-ABL-positive cells and potentiates imatinib mesylate effect. Basic Clin Pharmacol Toxicol 2013; 113: 103–112. 23551499 10.1111/bcpt.12073

[bib22] Hossain GS, Li J, Shin HD, Du G, Liu L, Chen J. L-Amino acid oxidases from microbial sources: types, properties, functions, and applications. Appl Microbiol Biotechnol 2014; 98: 1507–1515. 24352734 10.1007/s00253-013-5444-2

[bib23] Antonyuk VO, Klyuchivska OY, Stoika RS. Cytotoxic proteins of *Amanita virosa* Secr. mushroom: purification, characteristics and action towards mammalian cells. Toxicon 2010; 55: 1297–1305. 20153765 10.1016/j.toxicon.2010.01.023

[bib24] Utz PJ, Anderson P. Life and death decisions: regulation of apoptosis by proteolysis of signaling molecules. Cell Death Differ 2000; 7: 589–602. 10889504 10.1038/sj.cdd.4400696

[bib25] Green DR, Reed JC. Mitochondria and apoptosis. Science 1998; 281: 1309–1312. 9721092 10.1126/science.281.5381.1309

[bib26] Emam H, Zhao QL, Furusawa Y, Refaat A, Ahmed K, Kadowaki M et al. Apoptotic cell death by the novel natural compound, cinobufotalin. Chem Biol Interact 2012; 199: 154–160. 22898211 10.1016/j.cbi.2012.07.005

[bib27] Izidoro LF, Ribeiro MC, Souza GR, Sant'Ana CD, Hamaguchi A, Homsi-Brandeburgo MI et al. Biochemical and functional characterization of an L-amino acid oxidase isolated from *Bothrops pirajai* snake venom. Bioorg Med Chem 2006; 14: 7034–7043. 16809041 10.1016/j.bmc.2006.06.025

[bib28] Samel M, Vija H, Ronnholm G, Siigur J, Kalkkinen N, Siigur E. Isolation and characterization of an apoptotic and platelet aggregation inhibiting L-amino acid oxidase from *Vipera berus berus* (common viper) venom. Biochim Biophys Acta 2006; 1764: 707–714. 16574513 10.1016/j.bbapap.2006.01.021

[bib29] Torii S, Naito M, Tsuruo T. Apoxin I: a novel apoptosis-inducing factor with L-amino acid oxidase activity purified from Western diamondback rattlesnake venom. J Biol Chem 1997; 272: 9539–9542. 9083096 10.1074/jbc.272.14.9539

[bib30] Alves RM, Antonucci GA, Paiva HH, Cintra AC, Franco JJ, Mendonca-Franqueiro EP et al. Evidence of caspase-mediated apoptosis induced by L-amino acid oxidase isolated from Bothrops atrox snake venom. Comp Biochem Physiol A 2008; 151: 542–550. 10.1016/j.cbpa.2008.07.007 18804547

[bib31] Chen YC, Chen SY, Ho PS, Lin CH, Cheng YY, Wang JK et al. Apoptosis of T-leukemia and B-myeloma cancer cells induced by hyperbaric oxygen increased phosphorylation of p38 MAPK. Leuk Res 2007; 31: 805–815. 17064767 10.1016/j.leukres.2006.09.016

[bib32] Boatright KM, Salvesen GS. Mechanisms of caspase activation. Curr Opin Cell Biol 2003; 15: 725–731. 14644197 10.1016/j.ceb.2003.10.009

[bib33] Circu ML, Aw TY. Reactive oxygen species, cellular redox systems, and apoptosis. Free Radic Biol Med 2010; 48: 749–762. 20045723 10.1016/j.freeradbiomed.2009.12.022 PMC2823977

[bib34] Barbosa IA, Machado NG, Skildum AJ, Scott PM, Oliveira PJ. Mitochondrial remodeling in cancer metabolism and survival: potential for new therapies. Biochim Biophys Acta 2012; 1826: 238–254. 22554970 10.1016/j.bbcan.2012.04.005

[bib35] Ashkenazi A, Dixit VM. Death receptors: signaling and modulation. Science 1998; 281: 1305–1308. 9721089 10.1126/science.281.5381.1305

[bib36] Diaz GD, Li QJ, Dashwood RH. Caspase-8 and apoptosis-inducing factor mediate a cytochrome *c*-independent pathway of apoptosis in human colon cancer cells induced by the dietary phytochemical chlorophyllin. Cancer Res 2003; 63: 1254–1261. 12649185

[bib37] Zhang H, Yang Q, Sun M, Teng M, Niu L. Hydrogen peroxide produced by two amino acid oxidases mediates antibacterial actions. J Microbiol 2004; 42: 336–339. 15650691

[bib38] Kitani Y, Tsukamoto C, Zhang G, Nagai H, Ishida M, Ishizaki S et al. Identification of an antibacterial protein as L-amino acid oxidase in the skin mucus of rockfish *Sebastes schlegeli*. FEBS J 2007; 274: 125–136. 17140417 10.1111/j.1742-4658.2006.05570.x

[bib39] Lee ML, Tan NH, Fung SY, Sekaran SD. Antibacterial action of a heat-stable form of L-amino acid oxidase isolated from king cobra (*Ophiophagus hannah*) venom. Comp Biochem Physiol C 2011; 153: 237–242. 10.1016/j.cbpc.2010.11.001 21059402

[bib40] de Melo Alves Paiva R, de Freitas Figueiredo R, Antonucci GA, Paiva HH, de Lourdes Pires Bianchi M, Rodrigues KC et al. Cell cycle arrest evidence, parasiticidal and bactericidal properties induced by L-amino acid oxidase from *Bothrops atrox* snake venom. Biochimie 2011; 93: 941–947. 21300133 10.1016/j.biochi.2011.01.009

[bib41] Zhang L, Wu WT. Isolation and characterization of ACTX-6: a cytotoxic L-amino acid oxidase from *Agkistrodon acutus* snake venom. Nat Prod Res 2008; 22: 554–563. 18415865 10.1080/14786410701592679

[bib42] Kishimoto M, Takahashi T. A spectrophotometric microplate assay for L-amino acid oxidase. Anal Biochem 2001; 298: 136–139. 11673909 10.1006/abio.2001.5381

[bib43] Pislar AH, Zidar N, Kikelj D, Kos J. Cathepsin×promotes 6-hydroxydopamine-induced apoptosis of PC12 and SH-SY5Y cells. Neuropharmacology 2014; 82: 121–131. 23958447 10.1016/j.neuropharm.2013.07.040

[bib44] Gobec M, Obreza A, Prijatelj M, Brus B, Gobec S, Mlinaric-Rascan I. Selective cytotoxicity of amidinopiperidine based compounds towards Burkitt's lymphoma cells involves proteasome inhibition. PLoS One 2012; 7: e41961. 22860040 10.1371/journal.pone.0041961 PMC3408433

[bib45] Hafner A, Obermajer N, Kos J. Gamma-enolase C-terminal peptide promotes cell survival and neurite outgrowth by activation of the PI3K/Akt and MAPK/ERK signalling pathways. Biochem J 2012; 443: 439–450. 22257123 10.1042/BJ20111351

