# Supplementary figures and images for: Cytotoxic L-amino-acid oxidases from Amanita phalloides and Clitocybe geotropa induce caspase-dependent apoptosis
Source: Cell Death Discov. 2016 Mar 21;2:16021–. doi: 10.1038/cddiscovery.2016.21 (PMC4979486; doi:10.1038/cddiscovery.2016.21)

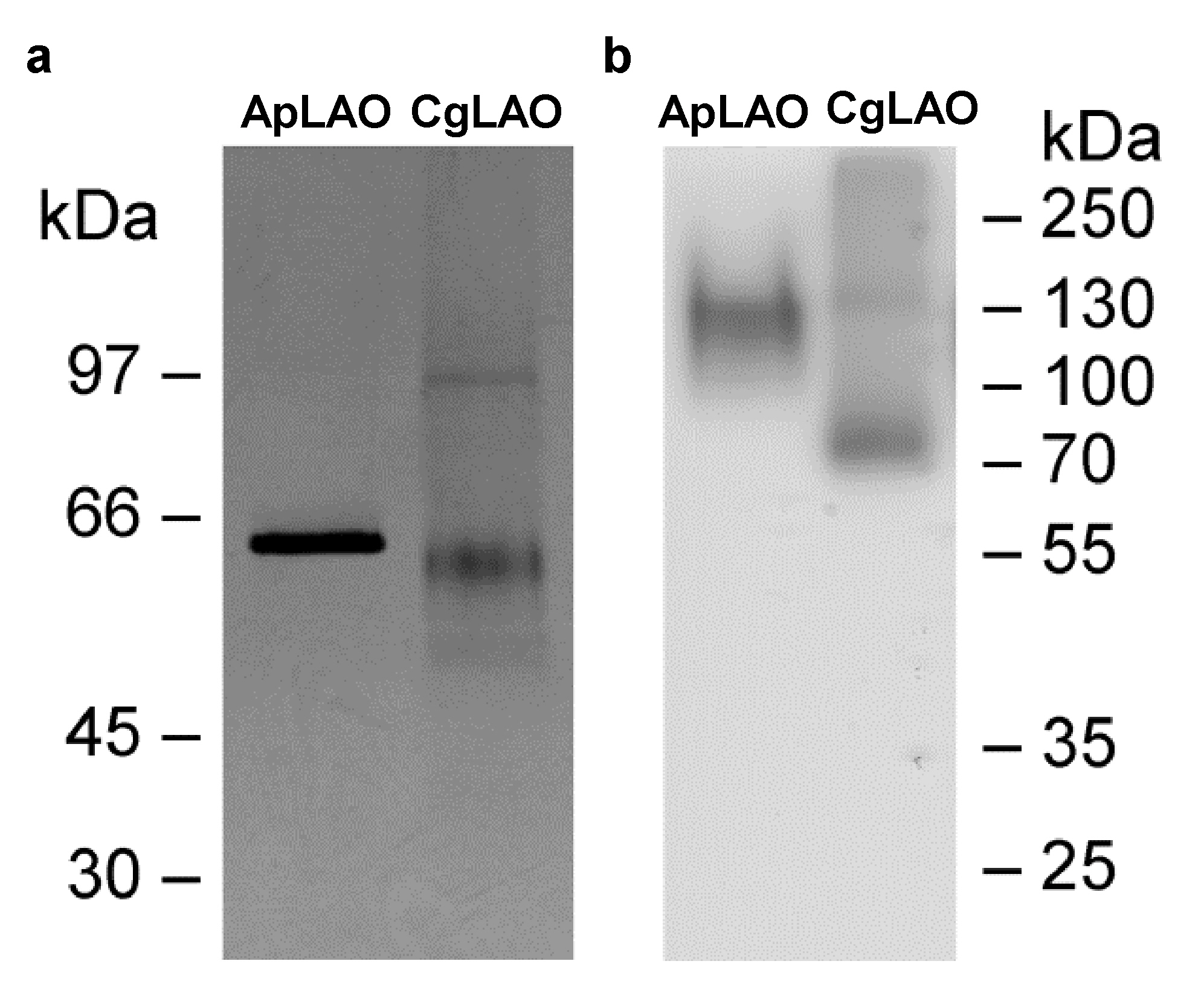

Supplement: Supplementary Figure S1 [file cddiscovery201621-s1.jpg]
