# Supplementary material for: Cytotoxic L-amino-acid oxidases from Amanita phalloides and Clitocybe geotropa induce caspase-dependent apoptosis
Source: Cell Death Discov. 2016 Mar 21;2:16021–. doi: 10.1038/cddiscovery.2016.21 (PMC4979486; doi:10.1038/cddiscovery.2016.21)
Supplement: Supplementary Figure legend [file cddiscovery201621-s2.pdf]

Figure legend for Supplementary data

Figure S1. Cytotoxic proteins isolated and purified from *A. phalloides* and *C. geotropa*. (**a, b**) SDS-PAGE (**a**) and in-gel LAO activity (**b**) analyses of purified ApLAO and CgLAO. (A) Purified ApLAO and CgLAO under denaturing conditions in a 10 % polyacrylamide gel visualized by silver staining. (B) Following SDS-PAGE under non-denaturing conditions in a 10 % polyacrylamide gel, LAO activity was detected by staining for H_2O_2 production for 1 h using the *o*-phenylenediamine - horseradish peroxidase system and 5 mM L-Leu as substrate in 0.1 M Bis-Tris, pH 5.5.
